# Clinical analysis of distribution and drug resistance of pathogenic bacteria in blood culture of Dalian Municipal Central Hospital from 2015 to 2019

**DOI:** 10.12669/pjms.38.7.5377

**Published:** 2022

**Authors:** Jinghua Gao, Jing Song

**Affiliations:** 1 Jinghua Gao, Department of Clinical Laboratory, Dalian University Affiliated Xinhua Hospital, Dalian 116021, Liaoning, China; 2 Jing Song Department of Clinical Laboratory, Dalian Municipal Central Hospital, Dalian 116033, Liaoning, China

**Keywords:** Blood culture, Pathogenic bacteria, Drug resistance

## Abstract

**Objectives:**

To analyze the distribution of common pathogenic bacteria and pattern of drug resistance in the blood culture of inpatients.

**Methods:**

This was a descriptive study. Blood culture data of inpatients of Dalian Municipal Central Hospital from January 2017 to December 2020 were collected from microbiology laboratory for retrospective analysis.

**Results:**

A total of 24,786 specimens were submitted for examination from inpatients from 2015 to 2019, and 2131 strains of clinically non-repetitive pathogenic bacteria were identified. There were 1135 G-positive cocci (53.26%), including 248 strains of *Staphylococcus* *hominis* (21.85%) and 68 strains of *Streptococcus species* (5.99%). Other G-positive cocci 8 strains (0.70%). G-positive cocci were most sensitive to datomycin, linezolid and vancomycin. There were 923 G-negative bacilli (43.31%), including 476 strains (51.57%) of *Escherichia coli*, 244 strains (26.44%) of *Klebsiella pneumoniae* and 130 strains (14.08%) of *Acinetobacter baumannii*. G-negative bacilli were most sensitive to amikacin. Most of the blood specimens were obtained from the ICU patients (42.98%) followed by nephrology (8.68%) and respiratory medicine (7.32%).

**Conclusion:**

G-positive bacteria were mainly detected in the positive blood culture samples of inpatients in this hospital. Daptomycin, linezolid and vancomycin were preferred for G-positive cocci, while amikacin was highly sensitive to G-negative bacilli.

## INTRODUCTION

Bloodstream infection is a serious systemic infectious disease, which refers to pathogenic microorganisms invade the blood circulation, reproduce in the blood, produce and release toxins and other metabolites, induce the release of cytokines, thus causing systemic infection, poisoning and systemic inflammatory reaction.^1^ As the gold standard for diagnosing bloodstream infection, blood culture can provide accurate and reliable basis for clinical diagnosis of bloodstream infection and rational use of antibiotics.^2^

Antimicrobial agents are the preferred treatment for infectious diseases whose extensive development and wide application will lead to their own gradually reduced susceptibility to pathogens.^3^ Antimicrobial resistance (AMR) has an increasing impact on clinical treatment and has become a worldwide public health problem.^4^ Studies have shown that inappropriate empiric antimicrobial therapy is an independent risk factor for increased mortality, mostly in patients with blood infections caused by Staphylococcus aureus or enterobacter.^5^ Furthermore, different diseases may differ greatly in their pathogenic bacteria and drug resistance due to region and time, which in turn affects the treatment outcome.^6^

In this study, a retrospective analysis was conducted on the principal pathogenic bacterial species and their drug resistance patterns in blood culture of inpatients in Dalian Municipal Central Hospital from 2015 to 2019, to provide a reference for clinicians in the use of antibiotics and the formulation of refractory infection control plans.

## METHODS

The study a retrospective descriptive study was approved by the Institutional Ethics Committee of Dalian University Affiliated Xinhua Hospital on May 5, 2019 (No. [2019]052).

### Inclusion criteria

Blood culture specimens of inpatients in Dalian Municipal Central Hospital from January 2015 to December 2019.

###  Exclusion criteria

The duplicate strains and suspected contaminating bacteria from the same case.

All the instruments used, including French Mérieux BacT/ALERT 3D 480 automatic blood culture instrument and supporting blood culture bottles, French Mérieux VITEK 2 Compact automatic microbial analysis system, Columbia blood agar/Eosin Methylene blue separation agar plate, M-H agar plate, etc. were purchased from Zhengzhou Barrett Biotechnology Liability Company. Various quality control strains, including *Staphylococcus* *aureus* (ATCC29213), *Escherichia* *coli* (ATCC 25922), *Pseudomonas* *aeruginosa* (ATCC 27853), and *Enterococcus* *faecalis* (ATCC 29212), were provided by the Clinical Laboratory Centre of the Ministry of Health.

All blood culture results from January 2015 to December 2019 were retrieved from the pathogenic microbiology Laboratory system database of our hospital. The specimen collection process during this period was as follows: Venous blood specimens from both sides of patients with systemic infection were collected according to aseptic procedures and injected into aerobic and anaerobic bottles respectively. After full mixing, the culture bottles were placed in the automatic blood culture instrument. Specimens that did not report positive results after six days of culture were judged to be negative. In case of a positive alarm of aerobic bacteria, the specimens should be transferred to sterile growth in time and cultured in a 35°C 5% CO_2_ culture environment. In case of a positive alarm of anaerobe bacteria, the specimens should be transferred to sterile growth and put into anaerobic bags, and then cultured in an anaerobic environment. Strains were identified by Gram staining and VITEK 2 Compact automatic microbial analysis system was utilized to perform strain identification and drug susceptibility test *in vitro* on the isolated single colonies. All operations were carried out in strict accordance with the requirements of the 4th edition of the “National Guide to Clinical Laboratory Procedures”, and the results are judged by referring to the M100-S31 document of the Clinical and Laboratory Standards Institute (CLSI).^7^

### Statistical Analysis

WHONT2020 software was used for drug sensitivity analysis, and the data were exported to calculate the ratio in an Excel list.

## RESULTS

A total of 24,786 clinical specimens were submitted for examination from inpatients from January 2015 to December 2019, and 2131 strains of clinically non-repetitive pathogens were identified with a positive detection rate of 7.16%. The highest positive rate of pathogenic bacteria was found from the samples obtained in EICU (Emergency Intensive Care Unit), where 407 non-repeated pathogens were detected in 308 positive samples, accounting for 19.1% of the total detected strains and it was followed by MICU (Adult Intensive Care Unit), where 289 non-repetitive pathogens were detected in 239 specimens, accounting for 13.56%.

Among the 2131 strains of clinically non-repetitive pathogens detected. There were 1135 G-positive cocci (53.26%), including 248 strains of *Staphylococcus* *hominis* (21.85%) and 68 strains of *Streptococcus species* (5.99%). MICU and EICU were the departments with the highest detection rate, with 53 strains of *Staphylococcus* *hominis* each being detected. And there were 923 G-negative bacilli (43.31%), 476 strains (51.57%) of *Escherichia* *coli*, 244 strains (26.44%) of *Klebsiella* *pneumoniae*, 130 strains (14.08%) of *Acinetobacter* *baumannii*, 32 strains (3.47%) of *Enterobacter* *cloaca*, 30 strains (3.25%) of *Pseudomonas* *aeruginosa* and 11 strains of other G-negative bacilli were detected. Among the G-negative bacilli detected in the highlighted departments, there were 243 cases of *Escherichia* *coli*. MICU was the department with the highest detection rate, with 37 strains being detected (Table-I).

**Table I:** Distribution of pathogens in the blood samples obtained from various hospital departments (2015 to 2019).

Pathogenic bacteria (%)	SICU (152 strains)	MICU (281 strains)	EICU (385 strains)	NICU (63 strains)	Surgery (195 strains)	Internal Medicine (626 strains)
G-positive cocci (53.26)	75 (49.34)	183 (65.12)	227* (59.48)	54 (85.71)	86 (44.62)	292 (52.56)
*Enterococcus* *faecalis*	4 (2.63)	1 (0.36)	7* (1.82)	0 (0)	5 (2.56)	7 (1.12)
*Enterococcus* *faecium* (n=114)	15 (9.87)	25 (8.90)	41* (10.65)	3 (4.76)	9 (4.62)	21 (3.35)
*Staphylococcus* (n=68)	6 (3.95)	12* (4.27)	8 (2.08)	3 (4.76)	16 (8.21)	23 (1.67)
*Salmonella para-typhi C*	6 (3.95)	6 (2.14)	14* (3.64)	5 (7.94)	2 (1.03)	13 (2.08)
*Staphylococcus* *aureus* (n=217)	17 (11.18)	50 (17.79)	57* (14.81)	15 (23.81)	20 (10.26)	58 (9.27)
*Staphylococcus* *haemolyticus* (n=84)	5 (3.29)	21 (7.47)	22* (5.71)	5 (7.94)	6 (3.08)	25 (3.99)
*Staphylococcus* *hominis* (n=248)	17 (11.18)	53* (18.86)	53* (13.77)	17 (26.98)	18 (9.23)	90 (14.38)
Other	5 (3.29)	15 (5.34)	25 (6.49)	6 (9.52)	10 (5.13)	55 (8.79)
G-negative bacilli (43.31)	77 (50.66)	98 (34.88)	158* (40.52)	9 (14.29)	109 (55.38)	334 (47.44)
*Acinetobacter* *baumannii* (n=93)	16 (10.53)	14 (4.98)	40* (10.39)	2 (3.17)	7 (3.59)	14 (2.24)
*Enterobacter* *cloacae* (n=69	10 (6.58)	2 (0.71)	6 (1.56)	0 (0)	2 (1.03)	49** (7.83)
*Escherichia* *coli* (n=243)	12 (7.89)	37* (13.17)	34 (8.83)	1 (1.59)	54 (27.69)	105 (16.77)
*Klebsiella acidophilus*	3 (1.97)	1 (0.36)	2 (0.52)	0 (0)	1 (0.51)	12** (1.92)
*Klebsiella* *pneumoniae* (n=196)	26 (17.11)	33 (11.74)	51* (13.25)	4 (6.35)	33 (16.92)	49 (7.83)
*Pseudomonas aeruginosa*	3 (1.97)	3 (1.07)	3 (0.78)	0 (0)	5 (2.56)	10*** (1.60)
*Salmonella* *enteritis*	0 (0)	0 (0)	0 (0)	0 (0)	0 (0)	19** (3.04)
Other	7 (4.61)	8 (2.85)	22 (5.71)	2 (3.17)	7 (3.59)	76 (12.14)

Among 1135 strains of G-positive cocci, a total of 113 strains of *Staphylococcus* *aureus*, which were of significant importance for clinical treatment, were detected, including 71 strains of methicillin-resistant *Staphylococcus* *aureus* (MRSA) and 42 strains of Methicillin-sensitive *Staphylococcus* *aureus* (MSSA). Among coagulase-negative staphylococcus, 647 strains of *methicillin-resistant coagulase-negative staphylococcus* (MRSCNS) and 143 strains of *methicillin-sensitive coagulase-negative staphylococcus* (MSCNS) were detected. no *staphylococci* were found to be resistant to daptomycin, linezolid, and vancomycin. MSSA was more sensitive to most antibiotics, except for floxacin antibiotics (ciprofloxacin, levofloxacin, and moxifloxacin), and its resistance rate to floxacin antibiotics was about 30%, slightly higher than that of MRSA (20%). Both *Enterococcus* *faecium* and *Enterococcus* *faecalis* were highly resistant to penicillin G and sensitive to daptomycin, linezolid, and vancomycin. No significant changes in the resistance of *Staphylococcus species* from 2015 to 2019 were seen. The detection rate of coagulase-negative staphylococcus was significantly lower in 2017-2019 than in 2015 and 2016 (Table-II and Table-III).

**Table II:** Resistance of important G-positive cocci to commonly used antibacterial drugs from 2015 to 2019.

Name of antibiotics	Staphylococcus aureus (n=113)				Coagulase-negative staphylococcus				Enterococcus faecium (n=114 strains)		Enterococcus fae2calis (n=24 strains)	

	MRSA (71 strains)		MSSA (42 strains)		MRSCNS (647 strains)		MSCNS (143 strains)					

	R	S	R	S	R	S	R	S	R	S	R	S
Penicilli G	100	0	92.7	7.3	100	0	79.3	20.7	99.2	0.8	100	0
Oxacillin	100	0	0	100	100	0	0	100				
Gentamicin	19.7	78.9	9.5	90.5	55.6	38.6	11.9	85.3				
Rifampicin	2.8	95.8	0	100	7.3	92.1	1.4	97.9	73.4	17.7	28.1	46.9
Ciprofloxacin	25.4	54.9	33.3	50	75.7	23	17.5	81.1	89.5	4.8	21.9	65.6
Levofloxacin	20.6	75	31.6	68.4	58.8	26.1	10	83.6	89.7	8.6	23.3	73.3
Moxifloxacin	21.1	76.1	31	69	57.7	24.9	10.5	82.5				
Cotrimoxazole	3	97	0	100	50.8	49.2	29.1	70.9				
Clindamycin	49.3	47.9	28.6	69	47.8	49	14	83.2				
Daptomycin	0	100	0	100	0	100	0	100	0	100	0	100
Erythromycin	78.9	19.7	59.5	40.5	89.5	9.6	72.7	27.3	93.5	1.6	71.9	18.8
Linezolid	0	100	0	100	0	100	0	100	0.8	99.2	0	100
Vancomycin	0	100	0	100	0	100	0	100	2.4	97.6	0	100
Tetracyclin	19.7	78.9	9.5	90.5	37.2	60.9	21	79	72.6	26.6	90.6	9.4
Ampicillin									88.7	11.3	0	100

R: Resistance rate (%); S: Sensitivity rate (%).

**Table III:** Resistance rate of main Staphylococcus sp. bacteria to commonly used antibacterial drugs from 2015 to 2019 (%).

	Staphylococcus aureus (113 strains)					Coagulase-negative staphylococcus (790 strains)				

Name of antibiotics	2015	2016	2017	2018	2019	2015	2016	2017	2018	2019

	15 strains	25 strains	19 strains	25 strains	29 strains	202 strains	201 strains	123 strains	138 strains	126 strains
Penicillin G	92.9	92	100	100	100	95	95	96.5	96	94.1
Oxacillin	46.7	64	63.2	72	62.5	86.6	85.6	77.2	77.2	78.6
Gentamicin	13.3	20	15.8	20	12.5	51	45.3	55.3	47.1	41.3
Rifampicin	0	4	0	0	3.1	6.4	4	5.7	9.6	7.1
Ciprofloxacin	33.3	40	21.1	16	34.4	67.8	66.2	61.8	66.2	63.5
Levofloxacin	21.4	28	21.1	15.8	34.4	52.5	45.8	51.2	47.8	54
Moxifloxacin	20	32	21.1	16	34.4	52	45.3	47.2	48.5	54
Clindamycin	20	44	68.4	48	28.1	43.6	32.8	54.5	46.3	34.9
Daptomycin	0	0	0	0	0	0	0	0	0	0
Erythromycin	66.7	68	73.7	76	71.9	85.6	85.6	86.2	84.6	92.1
Linezolid	0	0	0	0	0	0	0	0	0	0
Vancomycin	0	0	0	0	0	0	0	0	0	0
Tetracyclin	20	16	26.3	12	9.4	36.6	32.3	30.1	36.8	34.1
Cotrimoxazole				0	3.1				53	39.7

Among 923 strains of G-negative bacilli, Amikacin showed favorable antibacterial activity against *Escherichia* *coli*, *Klebsiella* *pneumoniae*, *Enterobacter* *cloacae* and *Pseudomonas* *aeruginosa*, with a sensitivity rate of >90%. Carbapenem antibacterial drugs had the optimal antibacterial activity against *Escherichia* *coli*, with a resistance rate of <2%, and also had high antibacterial activity against *Klebsiella* *pneumoniae*, with a drug resistance rate of < 10%; *Enterobacter* *cloacae* was not sensitive to carbapenems, with a drug resistance rate as high as 100%. In addition to amikacin, cefoperazone combined with sulbactam showed good antibacterial activity, with a sensitivity rate of > 80%; *Pseudomonas* *aeruginosa* was also insensitive to carbapenems, with a sensitivity of > 90% to tobramycin. *Acinetobacter* *baumannii* was highly sensitive to minocycline (84%) but insensitive to other commonly used antibacterial drugs (< 25%) (Table-IV).

**Table IV:** Resistance of major G-negative bacilli to commonly used antibacterial drugs.

Name of antibiotics	Escherichia coli (243 strains)		Klebsiella pneumoniae (196 strains)		Acinetobacter baumannii (103 strains)		Enterobacter cloacae (69 strains)	

	R	S	R	S	R	S	R	S
Ampicillin	87.4	12.6	100	0				
Piperacillin	73.8	20.2	56	36.8	84.6	10.3	58.8	41.2
Amoxicillin/Clavulanate	6.2	76	17.3	65.4				
Cefoperazone/Sulbactam	4.6	88.4	19.3	72.8	41.6	24.8	12.5	84.4
Ampicillin/Sulbactam	50.2	28.4	43.1	48.9	84.6	7.7		
Ticarcillin/Clavulanate	4.8	84.2	19.2	66.3	82.1	17.9	40	40
Piperacillin/Tazobactam	3.2	94.3	18	78.7	85.2	11.1	21.9	75
Cephazolin	59.2	40.8	46.5	53.5				
Cefuroxime	50	45.7	40.7	55.1				
Ceftazidime	25.9	68.9	24.3	71.6	90.9	6.8	30	70
Ceftriaxone	50.4	49.5	36.5	62.7	95.1	4.9	31.2	68.8
Cefotaxime	51.1	48.8	40.6	1.4	100	0	28.6	71.4
Cefepime	37.5	60.7	25	72.1	89.3	9.7	18.8	75
Cefotetan	1.7	97.5	11.4	86.7				
Cefoxitin	6.6	85.1	20.1	75.5				
Aztreonam	40.4	59	30.7	64.9			29	71
Ertapenem	1.3	98.7	8.6	91.4			0	100
Imipenem	0.3	99.4	10.7	89.3	87.5	12.5	0	100
Meropenem	0.6	99.4	10.7	89.3	85.7	14.3	0	100
Amikacin	2.9	96	9.8	90.2	73.7	25	3.1	96.9
Gentamicin	49.9	49.3	28.7	69.7	86.4	13.6	15.6	71.9
Tobramycin	31	48	18.4	68.9	83.5	13.6	28.1	71.9
Ciprofloxacin	63.9	33	30.3	68	89.3	10.7	21.9	71.9
Levofloxacin	60.2	36.6	26	70.2	83.9	16.1	17.9	78.6
Cotrimoxazole	64.3	35.7	30	70	75	25	33.3	66.7
Tetracyclin	62.3	37	46.2	51.9	82.1	17.9	53.3	40
Tigecycline					1.6	11.3		
Minocycline					4	84		

## DISCUSSION

Bloodstream infection (BSI) is a systemic infectious disease caused by the invasion of pathogenic microorganisms such as bacteria and fungi into the blood stream.^8^ In recent years, with the continuous increase of invasive operations and irrational use of broad-spectrum antibiotics and corticosteroids, the incidence and mortality of BSI have been increasing year by year.^9^ About 31 million cases of sepsis occur globally each year.^10^ At present, blood culture is still the gold standard for diagnosing bloodstream infection. A total of 24,786 specimens were collected in this study, and 2131 strains of pathogenic bacteria were isolated and cultured, with a positive rate of 7.15%. G-positive cocci were the main positive strains, contrary to the results of Mao S et al.^11^, which may be related to the difference in specimen quantity, geographical location, and living habits. However, the results of this study were similar to Meidrops K et al.^12^, which may be related to the difference in the distribution of pathogenic bacteria in different departments.

Antibiotics are the first choice for clinical treatment of bloodstream infection, but because of the abuse of antibiotics, the drug resistance of pathogenic bacteria is increasingly serious.^13^ Therefore, it is of great significance to carry out blood culture and drug sensitivity tests of pathogenic bacteria in early-stage to clarify the distribution and drug resistance of pathogenic bacteria to improve the rationality of drug use and control the development of the disease.^14^ In this study, 407 positive strains were detected in EICU, accounting for 19.1% of the total positive strains, followed by MICU, with a total of 289 strains (13.56%) detected. Among the pathogenic bacteria detected in EICU, 57 strains of *Staphylococcus* *epidermidis* were highly resistant to penicillin G. No strains resistant to daptomycin, linezolid, and vancomycin were detected, which was similar to the reported drug resistance of *Staphylococcus* *epidermidis*.^15^-^17^

A total of 317 strains of *Escherichia* *coli* were detected in the positive specimens, accounting for 14.88% of the total positive strains. The positive specimens were mainly concentrated in MICU (37 strains), EICU (34 strains), and Internal Hematology (32 strains), with a sensitivity rate of > 99% to imipenem and meropenem. This may be because medical patients mainly involve urinary tract infections or digestive tract infections. In patients with complex urinary tract infections, meropenem has been shown to have an advantage at the compound end point of clinical cure or improvement and microbial eradication.^18^ A total of 206 pathogenic bacteria were detected in Surgery, mainly *Escherichia* *coli* (54 strains) and *Klebsiella* *pneumoniae* (33 strains), with multi-drug resistance, which was similar to the results in the literature.^19^ Hepatobiliary Surgery detected the most positive strains (39 strains), while General Surgery detected only two positive strains, both of which were *Klebsiella* *pneumoniae*. Surgical inpatients were mostly surgery patients, so the prophylactic utilization of antibiotics may be the reason for the low positive detection rate and infection rate.^20^ In this study, the main pathogens detected in the medical specimens were Escherichia coli. The most pathogenic bacteria were detected in Nephrology (185 strains), mainly *Enterobacter cloacae* (44 strains), followed by Respiratory Medicine (156 strains), mainly *Staphylococcus* *hominis* (43 strains). Significant differences were found in the number of positive detections and main pathogenic bacteria detected among the departments.

### Limitations of this study

We only analyzed and discussed the cases included in our hospital, which may not be representative enough. We look forward to a multi-center study in the future to reach more comprehensive conclusions.

## CONCLUSION

G-positive bacteria were predominant in blood culture surface specimens of our hospital. Daptomycin, linezolid, and vancomycin are preferred for the treatment of G-positive cocci infections, and amikacin is highly sensitive in the treatment of G-negative bacilli. It is of great significance to pay close attention to the flora distribution, drug resistance and drug resistance change of bloodstream infection, guide the rational use of antibiotics and early control of infection, so as to prevent and control the outbreak of drug-resistant strains.

### Authors’ Contributions:

**JG:** Designed this study,prepared this manuscript, are responsible, accountable for the accuracy and integrity of the work.

**JS:** Collected and analyzed clinical data.
